# Political reconfiguration in the social space: data analysis and future perspective

**DOI:** 10.3389/fsoc.2023.1226509

**Published:** 2024-04-16

**Authors:** Daniele Battista

**Affiliations:** Department of Political and Social Studies, University of Salerno, Fisciano, Italy

**Keywords:** social networks, digital space, public engagement, controversial relationship, consensus-building

## Abstract

This paper aims to explore the complex and sometimes controversial relationship between social media and politics. The correlation between these two areas of research has always been less linear than a simplistic narrative might suggest, mainly because of the involvement of different scientific disciplines, such as sociology, political science, communication, social psychology and computer science. The decision to explore this topic is motivated by the persistent relevance of social media platforms in the current era. This growing centrality is also due to the accelerated digitization process that occurred during the pandemic phase in the digital ecosystem. In particular, the pandemic has contributed to a significant evolution of the concept of social networks. The methodology used is based on secondary data, and the work seeks to highlight the expansion of digital space resulting from the shift from a simple place of interaction to a digital space that reconfigured the mobilization and political action of Campania’s governor Vincenzo De Luca. In conclusion, the study reveals important findings on the president’s use of social media. For example, active citizen engagement strategies through direct interaction, timely information sharing, and mobilization of online resources are evident. Therefore, it becomes necessary to ask and understand whether the potential of new social technologies is being used to cultivate critical and massive citizen participation in democratic processes or whether it is being distorted for the sole purpose of increasing or consolidating consent.

## 1 Introduction

Nowadays, we are witnessing a new and multifaceted political landscape that demonstrates how the internet has become an extremely direct communication channel with the electorate, understood in a broader sense, as it is characterized by the use of more immediate language that allows online communicators to reinforce their image (Novelli, 2018). The times have passed when newspapers, as well as political rallies, represented the ultimate means of reaching essentially the militants and sympathizers of a particular political area. Currently, politicians are seen as figures who are close, everyday, and accessible, with whom the audience can identify (Bentivegna, 2014; De Rosa, 2014; Giansante, 2014). Thus, the internet is not only seen as a space useful for increasing the visibility and coverage of one’s content or for targeting opponents, but it represents much more (Vedel, 2005; Fuchs, 2009). From this rather intricate framework emerges an environment characterized by a repertoire of architectures and tools that serve the creation of meaning and the dissemination of value, rather than just unilateral communication tools as television and newspapers used to be considered (Annunziata, 2001). Digital media have also become a pervasive presence, and, thanks to mobile devices, they generate a phenomenon of permanent mediamorphosis that encompasses social reality, language, and culture (Castells, 2009).

For this reason, it is useful to embrace a basic assumption, namely the absolute awareness of being immersed, thanks to this deep intermixing, in a true and permanent election campaign (Blumenthal, 1980). Therefore, it is increasingly undeniable that politics, traditionally understood, is linked to its transposition into the digital media realm (Highfield, 2017), whose language is regulated by different principles. Social media offer an essential universe of debate, constituting an indispensable added value that political actors rely on and manage, akin to commercial logics (Foglio, 1999), with careful strategic approaches to achieve predetermined programmatic objectives. Leaders and professionals in the field take advantage of the Internet to bypass more traditional media and disseminate their message more quickly. In most cases, the goal is to promote media exposure, but in any case, this process underlies the broader process of disintermediation of politics, leading political figures to build their relationship directly and referentially with their electorate, bypassing traditional media narratives, at least exclusively (Parisi and Rega, 2010). Moreover, with social media, politics and its main actors do not address important political issues to indiscriminate audiences but rather tend to fulfill much broader electoral functions (Mazzoleni, 2021). In the current era, characterized by a continuous and perpetual campaign to build consensus in pre- and post-electoral phases (Palmieri, 2016), studying the differences in communication strategies implemented through social media by key political figures not only poses a challenge that takes into account an ever-evolving scenario in political communication but also holds undeniable relevance in highlighting old and new differences, more or less innovative strategies, or strategies that are truly suited to the digital landscape (Cepernich, 2017). In an era where political communication is increasingly dominated by digital media, the COVID-19 pandemic has represented a turning point in the relationship between politics and technology (Parsi, 2021). The increased use of social media has radically changed how politicians communicate with citizens. This phenomenon represents an increasingly significant field of study as political communication is becoming more influenced by algorithms, data analysis, and digital communication strategies (De Rosa, 2018). Moreover, the pandemic has underscored the need to better understand the role of digital media in democratic participation as most political activities have shifted online (Amoretti and Santaniello, 2021). During the lockdown period due to the COVID-19 pandemic, the use of video conferencing platforms such as Zoom, Microsoft Teams, and Google Meet became crucial in facilitating work activities, distance learning, and online meetings. According to data provided by the Agency for Digital Italy (AgID), there was a 500% increase in the use of these platforms in 2020 compared to the previous year. Additionally, social media played a significant role in keeping people connected during the lockdown. Platforms like Facebook, Instagram, and Twitter were used not only to maintain ties with friends and family but also to obtain updated information on restrictions and preventive measures against the virus. According to a report by We Are Social and Hootsuite, Italians spent an average of over 2 h per day on social media in 2020. These data contribute significantly to our understanding of the situation. In this sense, the contribution aims to analyze these phenomena and assess their impact on society, highlighting the case concerning Campania Governor Vincenzo De Luca. Furthermore, within the digital landscape that emerged during the pandemic period, new challenges and risks have also arisen for political processes and dynamics. There has been a growing interest in phenomena such as disinformation, propaganda, information warfare, hostile information campaigns, and informational disorder, commonly referred to as fake news. It is crucial to emphasize once again that digital media, which have increasingly assumed a central role in our daily lives, have accelerated a transition that has made the use of digital platforms essential for billions of individuals, transferring a wide range of educational, governmental, and healthcare services online. It should be noted that this transition has generated a contagious vortex, accompanied by an infodemic and a digital epidemic characterized by a flood of false information, undermining trust in science and verified sources in a period of great uncertainty (Radu, 2020). The pervasiveness of information deliberately created to deceive and mislead large groups of citizens (Wardle and Derakhshan, 2017) has drastically diminished trust in institutions, thereby influencing the digital ecosystem and jeopardizing the protection of fundamental rights. The magnitude of the damage caused online becomes even more evident in a context of ideological polarization and widespread propaganda that exploits the manipulation of public opinion, constantly testing digital resilience and democratic values (Terzis et al., 2020). This comprehensive overview further highlights the magnitude of the analyzed phenomenon and its broad scope within the surrounding context.

## 2 From governor to celebrity: the metamorphosis of Vincenzo De Luca

The phenomenon of Vincenzo De Luca as a celebrity leader represents a significant example of how media communication can become a determining factor in the success of a political leader. During the pandemic period, De Luca managed to emerge as a reference figure for many Italians thanks to his ability to effectively communicate through the media. His communication style is characterized using direct, incisive, and often ironic language that captures the audience’s attention and evokes strong emotions. His manner of presenting himself to the public has been described as strong and clear, aimed at being understood by the entire citizenry, often using dialectal terms to create a sense of closeness. Cutting irony is a distinctive feature of his interventions, particularly when announcing stringent restrictions that exceeded those of other Italian regions with a similar number of infection cases. During such announcements, he would use exaggerated epithets such as:

*“Uscirò con una mazza in mano, mi nasconderò dietro ai muri e comparirò non appena ne vedo uno: una botta in testa e lo lascio stecchito a terra.”*

*“I will come out with a club in hand, hide behind walls, and appear as soon as I see someone: a blow to the head and I’ll leave them flat on the ground.”*

This was the threat directed toward those venturing outside without a valid reason. Furthermore, during his participation in the television program “*Che tempo che fa*,” hosted by Fabio Fazio, he even referred to the host as a “big clod,” a term that immediately became a trending topic on Twitter. His rhetorical style, based on the ability to create vivid and memorable images, employs expressions and metaphors that resonate with the collective imagination. Furthermore, the former mayor of the city of Salerno presents himself as an authentic leader who is close to the people, speaking the language of ordinary individuals and knowing how to represent their concerns and issues. This empathetic dimension has been crucial in establishing an emotional connection with the public and consolidating his authority as a political leader during the pandemic period. Being a true media personality is also manifested through the skillful use of social media and traditional media, which have allowed him to reach a wide audience and maintain constant dialog with citizens. His videos on social networks have been hugely successful, going viral and generating high levels of user engagement. In summary, his figure represents an exemplary case of how media communication can be a determining factor in the success of a political leader. In just 25 s, much less than the “fifteen minutes of fame” envisioned by Andy Warhol as necessary to become famous in post-industrial society, De Luca rose to the role of a social star, transforming and genetically altering his political being into a “character,” a recognizable mask with distinct identity traits that make him unique. In fact, taking a step back, it was 20 March 2020, when Italy had been under lockdown due to the pandemic for just over 10 days, and the President of Campania, during his live broadcast on Friday afternoon on his Facebook profile, at the 29:50 mark, launched a strong attack against those who did not grasp the seriousness of the situation:

*“Mi arrivano notizie che qualcuno vorrebbe preparare la festa di laurea, mandiamo i carabinieri, ma li mandiamo con i lanciafiamme!.”*

*“I hear news that some people would like to organize a graduation party, let us send the police, but let us send them with flamethrowers!”*

It is perhaps from that Friday, from that precise moment when he threatens the young graduates who thought they could celebrate their coveted university degree with impunity, that the governor of Campania crosses the point of no return in traditional political communication and becomes, willingly or unwillingly, the object and subject of popular and populist communication. It is with that famous phrase, “I’ll send the police with flamethrowers,” that De Luca exchanges his 30-year history as a decisive politician and leader of Campania for entry into the realm of trend-setting politainment, guaranteeing him visibility that goes beyond the narrow regional borders. Thus, in the following months, the President of Campania repeatedly resorted to obvious lexical exaggerations, making an effort to tap into the plebeian imagination and employ rhetorical figures to align and shift the semantic register solely toward populism. The numbers from that post, for example, which were already skyrocketing within the first 24 h, remain impressive even after more than 3 years and have not ceased to rise. Not to mention our messaging chats that were literally invaded by De Luca memes and stickers of the Sheriff in all possible and imaginable versions. A flood of images and videos that, we could dare to say, fed on social networks and the internet continuously, almost pandemic-like.

## 3 Numbers and consensus building in digital politics

The methodology used involves the use of Fanpage Karma, which relies primarily on the analysis of quantitative data collected from social media. Therefore, it can be said that the use of these tools falls more within a quantitative research methodology. However, the analysis of quantitative data was still accompanied by an analysis that allowed for understanding the meaning and perception of the published content. This type of analysis can be considered an addition to quantitative research but does not necessarily represent a complete qualitative research methodology. Fanpage Karma primarily utilizes a data analysis-based methodology for market research and performance evaluation on social media. By collecting data from various social media platforms such as Facebook, Twitter, and Instagram, it provides analyses and reports on the reach, engagement, and impact of the content published on the governor’s various channels. Specifically, through a data analysis methodology based on the processing and interpretation of metrics such as the number of followers, reach, engagement, and user interaction with the content published on social media. By considering these metrics to measure the effectiveness of social media activities, we have certainly identified the strengths and weaknesses of the strategies implemented on different platforms. However, it is important to provide some clarifications within the broader context. With a few years of anticipation compared to the acceleration experienced during the pandemic, the interaction between political actors and citizens online has led to web politics (Mosca, 2012), promoting citizen involvement in public discourse and increased political engagement. This has surpassed the journalistic intermediation of traditional channels in terms of agenda-setting power (Mazzoleni and Bracciale, 2019), thanks to the interactivity allowed by digital media. However, there are also inherent limitations associated with their nature and exposure to possible manipulations and simplifications (Bentivegna and Boccia-Artieri, 2019). Particularly, the current political moment linked to the use of digital media in politics must confront an increased level of citizen participation and interaction online, generating effective communication strategies and actions in producing political messages that the public can appropriate, modify, and relay. Considering the pandemic as a triggering factor, the available data indicate that the current President of the Campania region has experienced significant success on social platforms, particularly on his Facebook page. Between March and December 2020, during the peak of the pandemic, the President’s Facebook page garnered over 15 million reactions, including likes, comments, and shares, and gained 772 thousand new followers.

**Figure 1:**
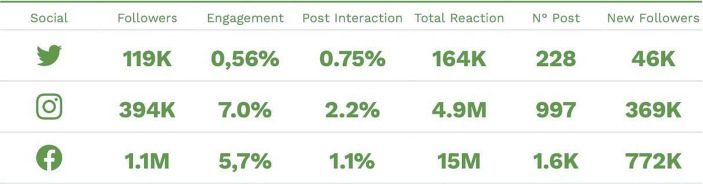
Scan conducted with Fanpage Karma between March and December 2020.

Additionally, it is interesting to note that the engagement rate, which represents the percentage of involvement in a single content posted on social media, is particularly significant for President De Luca, with a rate of 7% on Instagram and 5.7% on Facebook (Figure 1).

This evidence suggests the effectiveness of President De Luca’s online communication strategy during a global health crisis. However, for a more comprehensive evaluation of the success of his social media activities, it would be necessary to analyze additional factors, such as the type of content shared and its relevance to the target audience. The logic of traditional media sees the entry of new media logics into the current hybrid media ecosystem (Chadwick, 2013). These new media add new channels, formats, and languages to those of traditional media. President De Luca, despite initially expressing some hesitation toward the Chinese platform TikTok, has decided to embrace its popularity and join the platform. This choice seems to indicate an adaptation to the logics that govern the use of social media for political communication. After all, the use of TikTok is a strategy based on the ability to reach a young audience and influence them through short and engaging videos.

Although this platform has been subject to some controversies, it is undeniable that it offers an important communication channel to reach audience segments that are otherwise difficult to engage through traditional media (Battista, 2023). Yet, the governor had repeatedly criticized the excessive use of social media by national political figures. When asked about the choice of Lega Secretary Matteo Salvini to open his own TikTok profile, the President of the Campania Region expressed himself as follow.

*“Penso che siano tutte delle grandi palle, TikTok e Tic-tac. Rimango tenacemente convinto che alla fine contino i fatti, anche se inondi di fake news i social. Sappiamo tutti che, per l’80%, sui social compaiono palle, cose inventate, costruite a tavolino, algoritmi. Dietro i tweet non ci sono esseri umani ma algoritmi e soldi con i quali pagare gli algoritmi.”*

*“I think they are all a bunch of nonsense, TikTok and Tic-tac. I firmly believe that in the end, facts matter, even if social media is flooded with fake news. We all know that 80% of the time, what appears on social media are lies, things made up, constructed by algorithms. There are no human beings behind the tweets, only algorithms and money to pay for the algorithms.”*

However, it should not be underestimated that engaging with the audiences on these platforms, known as networked publics, can generate the risk of having messages and content debated or dismissed, reputation deteriorated or enhanced, and one’s proposal strengthened or defeated (Farrel and Drezner, 2008). Digital media, in fact, have expanded the reach of political expression and diverse publics, and the content between narratives and information comes from a variety of sources, not all of which are official channels. As a result, citizens and politicians interact with a vast volume of online material that the actors in the field inevitably have to manage. Access to information, previously available only to professionals, is now permitted to all citizens through the network, allowing them to inform themselves independently of traditional media and gather in independent groups, providing the online public sphere with the oversight prerogatives of democracy that were previously the prerogative of the press (Dahlgren, 2005). Even the role of gatekeeping (McQuail, 2012) has shifted from journalists in broadcast media to users, who have the capacity and possibility to produce and distribute news outside of mainstream channels. The newsworthiness of an event or news is conceived and disseminated by the network in the current moment. Thus, there is a transition from party democracy, intermediaries in favor of citizens, to public democracy (Grossi, 2011), based on a direct political and communicative link between leadership and citizens. The volume of opinions and dialog allowed by the network determines an increase in social capital, manifested in an increase in the collectivization of activism (Soha and Meyrowitz, 2016; Mazzoleni, 2021), access to collective information (Davis, 2016), and the quality and breadth of social relationships (Julien, 2015). These achievements are difficult to be limited by echo chambers and have led to significant upheavals and changes in society, even outside the network. Modes of engagement in public and political discourse evolve from collective action guided by a recognizable leadership figure to a connective action organized horizontally through networks (Bennett and Segerberg, 2013), but which is equally effective in inciting people to participate in physical demonstrations, regardless of the outcome (Coleman, 2016). Public opinion becomes a tool to reduce complexity, as Luhmann (1978) would say, a reality constituted by communicative processes that capture the attention of public opinion based on specific themes. In this way, we move from a traditionally complex and self-referential realm to an increasingly pop-political point, where public opinion becomes a territory of curiosity and social discourse outside the dynamics of classical politics, truly transformative and familiar. In the contemporary political-media space, the interdependence between social media and political communication, popular culture, and personalization allows for performing on the grand stage of democracy.

De Luca, during the pandemic, was able to channel all of this in his communicative trajectory. Moreover, if political communication during the coronavirus era was able to transform a prime minister into a sex symbol, thanks in part to the virality of the “Le bimbe di Conte” page, the De Luca phenomenon represented an explosive combination of personalization and the spectacularization of politics associated with emergency communication. From the first announcement of the extension of containment measures to the entire national territory, press conferences by the government, Civil Protection, health authorities, and local authorities filled the media agendas and transformed into true media events. The most unexpected effect of the quarantine was the increased popularity of press conferences among Italian citizens, who began to follow them regularly as a fixed appointment and adapted their viewing experience to the second-screen mode (Giglietto and Selva, 2014), meaning they commented and interacted with other viewers on social media. This phenomenon is comparable to the popularity of beloved television programs followed by viewers. Much was done by politicians and their staff to turn these conferences into real shows, and if there is a record in this sense, it can only be attributed to Vincenzo De Luca. With the live broadcasts from the official Facebook Page, the governor of Campania would have reached “numbers seen in US politics,” as reported by Il Corriere della Sera^1^ on their Instagram profile on 13 May 2020, quoting a member of the social media team. Every Friday at 3 p.m., during the press conferences of the Campania Region, hundreds of comments were received in real-time from Italian citizens. Among these, various categories of interventions can be distinguished. Firstly, there are those who seek clarifications on regional ordinances, expressing concerns about the restrictions imposed to address the health emergency. Secondly, there are also negative comments from haters, individuals who use social media to criticize and attack interlocutors without providing constructive contributions to the discussion. Finally, there are comments from citizens from other regions of Italy who express regret for not having a similarly authoritative governor in their own region or who hope for the President of the Region to have a national political career or access to high institutional positions. It is no secret that in times of crisis and uncertainty, humanity often seeks a strong leader who can provide a decisive and coherent response to the ongoing challenges. This principle is so universal that it can be considered a constant in human history. However, in the specific context we are analyzing, it is not appropriate to simplify and reduce the issue to a “rally around the flag” mechanism, which could be interpreted narrowly as a role of a savior of the nation (Moffitt, 2016). The popularity of the analyzed figure, in fact, represents one of the most prominent phenomena of the Covid-19 era, even appreciated by international personalities like top model Naomi Campbell. In particular, during the early weeks of quarantine, Campbell shared a video on Instagram featuring De Luca’s famous statement about flamethrowers being promised to those who violated restrictions during the lockdown, urging American policymakers to follow the example of the Italian governor.

This episode highlights how the figure of a strong and determined leader is able to generate wide attention, even at an international level, especially in the context of a global health emergency like the one we have experienced. This frenzied presence on platforms is characterized by political communication strategies that tend toward progressive polarization around the leader and intertwine with everyday life to bring the political actor closer to the voters (Bennett, 1998; Van Zoonen and Stanyer, 2013) and represent them as one of them through lifestyle politics (Giddens, 1997). The autonomous management of self-representation on social networks has limited the mediation of professionals and media apparatuses (Kruikemeier et al., 2016) as well as traditional parties (Vergeer and Hermans, 2013). Furthermore, regarding the electoral results of the latest political appointment in which the governor participated, it can be observed that the governor’s affiliated party, the Democratic Party, obtained the majority of votes in the elections, with a percentage of 16.9% and a total of 398,000 votes in favor of the list. However, it is important to note that the success of the Democratic Party was matched by the De Luca President list, which also garnered a considerable total of 313,000 votes, placing it second in terms of popularity among voters. These data demonstrate the relevance of De Luca’s figure within the regional political context and the effectiveness of his political branding strategy, which allowed his list to achieve significant results in the elections in question. Therefore, digital media have expanded the stage for political representation and the diverse publics, while the content comes from various sources within a continuum between storytelling and information. Thus, even in this context, there is a shift from the democracy of parties, which act as intermediaries for citizens, to the democracy of the public (Manin, 2013), based on a direct, political, and communicative link between leadership and citizens. Even De Luca exposes himself to the logic of representation, which is transformed into that of presentation, where the depth of analysis and debate typical of elected assemblies gives way to the pursuit of deliberative speed favored by the media (Sorice, 2009). The use of social media platforms and instant messaging as a means of communication with the public influences politics and electoral political processes (Rodrigues, 2020), as online participation is positively correlated with Internet usage but also positively correlated with offline participation (Vesnic-Alujevic, 2012). In fact, political messages that users receive through social networks, especially if they come from social networks with whom they are friends, can increase voting intentions (Kim and Min, 2015).

## 4 Politics continues to change its way of communicating

Social networks allow for expanding possibilities of participation and resource exchange, sharing emotional experiences both in interpersonal relationships and in broader cultural, social, and political contexts. Currently, they represent the dominant environment of political communication that unfolds in a predominantly mediated public space. Until the late 1990s, although there were variations in content delivery channels, all media were characterized by top-down content transmission by media organizations in different contexts. However, since the 2000s, political communication has undergone a revolutionary process. The discipline has penetrated unexpected fields and sectors, acquiring a protean nature that has significantly heightened the difficulty of interpreting cultural and communicative phenomena. In this inherently unstable scenario, social media have become crucial communication tools for politicians (Ceron, 2017; Stier et al., 2018). In fact, the use of Twitter and Facebook has even reached a saturation point in recent years (Tromble, 2018). Moreover, social media platforms allow politicians to reach a wide audience and communicate directly with voters (Vergeer and Hermans, 2013). A case that certainly represents a significant novelty in the complex and diverse landscape is the Chinese platform TikTok, known for hosting fast-paced and lighthearted content (Ballesteros Herencia, 2020), which has experienced an unprecedented surge in the past 2 years. However, despite content consumption, actual user engagement with political entertainment content, according to several studies (Berrocal-Gonzalo et al., 2014), remains limited. In today’s reality, the entire political and institutional sphere is permeated by the logic of media production and is subordinated to it when utilizing its channels. This current condition is identified as the mediatization of politics (Mazzoleni and Shulz, 1999). The effects that the media system has produced on the political system can be empirically observed and can be grouped into media effects and political effects. The former strongly highlights the phenomenon of spectacularization, that is, an increasing dramatization observable in political action, which has taken the form of pop politics in recent years (Mazzoleni and Sfardini, 2009). Additionally, the thematic construction of the political agenda becomes evident.

**Figure 2:**
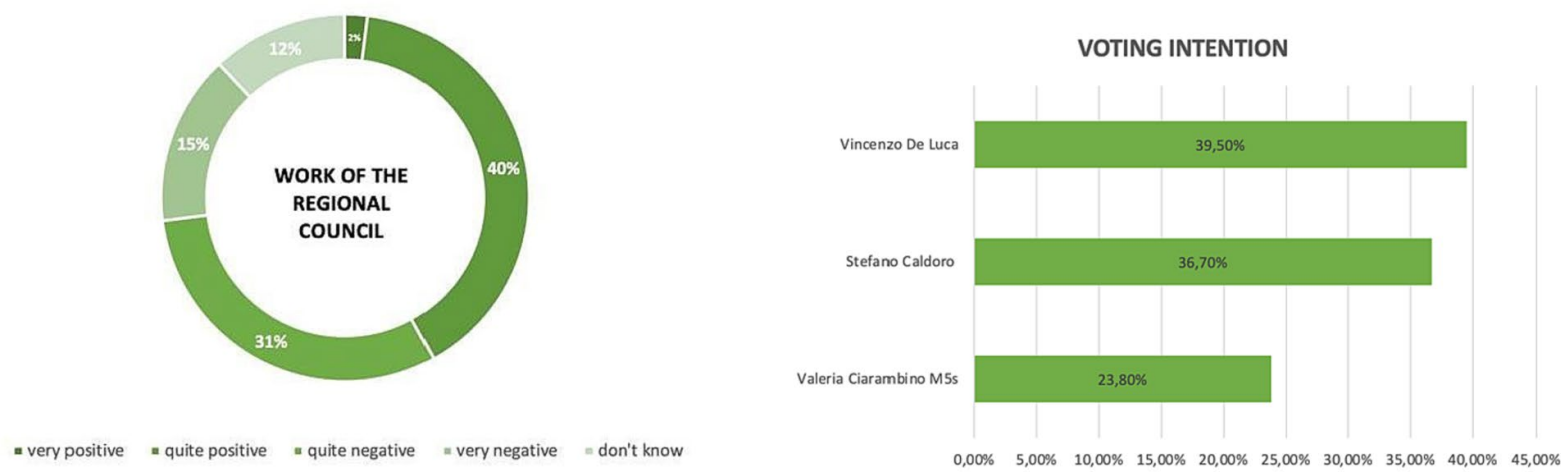
Approval rating and voting intentions conducted by Arcadia.com.

With the concept of Agenda Building (Semetko et al., 2013), the media compel politics to address specific issues. Another aspect resulting from media effects is undoubtedly the fragmentation of political discourse. This process, known as sound bites, arises from the reduction of media space devoted to political communication, which could lead to the trivialization of politics itself, as well as the perception of reality in general. Among the political effects, one that largely monopolizes the scene refers to the personalization of politics. A contamination with popular culture, understood in commercial terms, imparts distinct personal characteristics to politicians that go beyond the image of a party representative (Van Zoonen, 2005). Moreover, during the year 2019, the popularity of Vincenzo De Luca, the President of the Campania region, experienced a progressive decline (Figure 2).

This decrease in approval can be attributed to various factors and political dynamics that occurred during that period. The succession of scandals or controversies involving the governor may have compromised the trust and perception of citizens toward the regional president. The choices made in healthcare, infrastructure, or economic policies can have tangible effects on the daily lives of citizens and, consequently, on their evaluation of their leader. Finally, it is important to consider the broader political context in which this decline occurred. Throughout 2019, there were significant political events at the national level that may have influenced public opinion toward De Luca. For example, the European elections in May 2019 and the political tensions between the central government and regional governments could have contributed to an atmosphere of general mistrust toward politicians. Only later, De Luca emerged from this circumstance, where exacerbated by the pandemic effects, a strong democratic erosion occurred, contributing to the enhancement of the leader and a simultaneous downgrading of the role of parties, which experienced a considerable loss of trust (Dalton and Wattenberg, 2003), effectively replacing party democracy with public democracy (Manin, 2013). The governor positioned himself within an existing fracture. It is no coincidence that for years, some have argued (Calise, 2000) and identified a cause of the current scenario in the transition to an electoral system focused on candidates rather than parties. For example, in Italy, we find the personalization of politics triggered by changes in electoral laws (single preference, majority system, direct election of mayors and regional presidents), where the candidate’s name prevails over the party (Pasquino, 1990), and their statements take precedence over the political program, while physical appearance outweighs all other characteristics. Moreover, the concept of leadership is also discussed as a phenomenon linked to personalization that characterizes all modern Western democracies.

Hence, the need for a constant and no longer sporadic communication activity that encompasses the same rules of political communication. While on one hand, it can be a strategically indispensable appendage, on the other hand, it needs to be supported by a clear political design. Today, in the collective imagination, the law prevails that politicians must be charismatic, but this is not an automatic and simple process (Giorgino, 2020). It is therefore more important than ever to be able to convey the message, values, and distinctive traits that characterize the political profile, making it easily identifiable. In this historical period, social media platforms can represent a system based on a strong structure in which different strategies can become excellent allies in the growth or consolidation of political consensus. This state of affairs has also affected the President of Campania, who, with his speeches and political rhetoric, abandons the techno-institutional language (Dell’Anna, 2010). The latter is displaced by the media formats to which the public has become accustomed, namely entertainment, spectacle, and advertising. There are several causes attributable to the current issue, among which the television imaginary has certainly contributed to diversifying the role of politicians, politics, and the expectations placed on them by the public and voters (Couldry, 2008). In addition, the continuous delegitimization of traditional politics (Ceccarini and Diamanti, 2018) and the replacement of ideologized political elites with managerial elites (Crouch, 2003) should be mentioned. Moreover, nowadays, voters identify themselves less ideologically with traditional parties and are less cohesive among themselves, being more susceptible to emotional and personal persuasions (Sorice, 2011). In this context, the centrality of media representation emerges, which has prioritized the image of candidates over programs, projects, and political competences (Manin, 2010), and the subsequent massive use of political marketing that requires the involvement of professionals and consultants (Cacciotto, 2020). This condition desacralizes public discourse, adapting it to the standards of the media system within the framework of spectacularization. The paradigms of entertainment cross and hybridize the genres of information, politics, and political science, transforming the objects of entertainment or products of popular culture into infotainment.

All this encapsulates the result of seeking consensus through the adaptation of political language to that of the media and the attempt to highlight the personal side of politicians through the personalization of politics after its dramatization. The tendency toward personalization is inherent in all politics of modern democracies, while programs and parties lose importance (Mancini, 2011), and identification with the party is replaced by identification with the leader (Ceccobelli, 2017). The inclination toward this factor responds to the need to interest the floating electorate, which is poorly represented by parties but attracted to a leader seen as more intimately close. At this point, it is now the political leaders themselves who push for the personalization of politics, relying on their personal qualities of affability and likability, convinced that emotional consensus can overcome the resistance of an audience that is not attracted to formal politics. However, often the values conveyed deviate from those expected by the public or those exposed, causing a detachment to their detriment (Langer, 2010). The possibilities of interaction, association, and participation offered by digital media open the doors to a form of digital citizenship that can strengthen democratic processes and bring citizens closer to institutions through enhanced relationships. These potentialities could be grouped under terms such as interactivity, copresence, disintermediation, reduced costs, speed, and absence of boundaries (Bentivegna, 2002).

## 5 Conclusion

This contribution can be placed within the framework of a cross-sectional research as the topic is analyzed and addressed from various perspectives. The research is not limited to the digital world but extends to the sociological and political fields. This need is based on the parallel analysis of cultural phenomena from different social fields to grasp the factors that constitute the cause of differences in the structure and trend of events. The result of this work leads to defining a problem or improving knowledge in this regard. The reconstruction of the object of study through the consulted sources has proven useful in understanding its immense scope and complexity. Indeed, these changes could upheave and influence the functioning of democracy. It is evident that today’s reality has stored aversion toward new means of communication, but the question and concern mainly revolve around the relationship between consumption and civic engagement. The quality of political content and its reception by the public of citizens are of primary importance. In other words, how politics is represented in the spectacularized communication of politicians. Does it manage to generate interest, attraction, and participation among audiences, or does the digital turn with its populist implications create excitement without the personal commitment of citizens? On this topic, we find many conflicting results that make expectations about future progressions even more intriguing. Based on emerging findings, the fusion between politics and social media can play an increasing role in individual and collective styles of political practices and participation (Dahlgren, 2009). In fact, this combination can paradoxically represent a healthy contamination, even of simple political information, for broad segments of the public that are traditionally or intentionally distant and distracted from the political sphere. It is well-known that citizens with less interest in politics and lower attention to electoral campaigns benefit the most from the accidental encounter with political content on social media (Vaccari and Valeriani, 2021). Essentially, even so-called soft news, often considered ideal for an audience less accustomed to political life, represent the only source of information for millions of viewers. They contain just enough substantive information to prevent viewers from becoming completely estranged from the realm of political events. The point is to consider this novelty as an ally of participation rather than an obstacle. On the other hand, in an era like the present, where the use of digital platforms increasingly influences various sectors of society (Van Dijck et al., 2018), it becomes a pressing need for the political galaxy to find a compromise with media logic and secure the increasingly fragmented attention of the public of citizens. At this point, the popularization of politics should not be seen as a regression in the quality of the political message, but rather as a quest for greater appeal, ease of understanding, and the ability to reach even where the space for politics is limited or nonexistent. Digital media has become an important subject of study, as evidenced by the growth of various related fields of research. However, it is not only a research topic but has significantly influenced the social sciences in general. The approach used reflects the principles of digital positivism and projects toward a paradigm shift toward criticism of the digital realm. The advancement of such a model represents a material issue whose solution requires not only attitude changes but also institutional and political transformations, as well as a shift in the programmatic vision of investments. The process we are witnessing, far from being synonymous with the degradation or decline of politics, proves to be a necessary response to the transformation of society toward increasingly commercialized forms of communication. In the various social media platforms that prevail globally, where messages, channels, and audiences are increasingly fragmented and transient, the increasingly strong relationship between social and politics can be a valuable resource capable of reconciling the public with political institutions and reaching broader segments of citizens who are distant from politics. If this were the outcome, in the coming years, we could assert that political communication through social media will have proven to be a tool for radical renewal of awareness and of great social, democratic, and educational potential. And if the future inexorably pushes toward ever more liquid information, it is up to communication in this case to ensure a form of solid community. The analysis of the correlation between social media and politics is a complex and controversial topic that requires the integration of different scientific disciplines. The increasing centrality of social platforms in the current era has been amplified by the pandemic and the consequent acceleration of the digitization process. In particular, the transition from a simple place of interaction to a digital space has reconfigured the mobilization and political action of figures like the Governor of Campania, Vincenzo De Luca. In conclusion, the relationship between social media and politics is complex and multidisciplinary, but it remains crucial to understand how new social technologies can be employed ethically and responsibly in order to promote inclusive and conscious democratic participation. It would be interesting to explore the effects of political campaigns on social media, including communication strategies and political polarization. Additionally, analyzing the spread of disinformation and fake news on social media, along with policies to counteract them, could provide valuable insights into ensuring accurate and reliable information during elections and periods of crisis. At the same time, it is crucial to consider the implications of privacy and data security in the use of social platforms in the political context. However, it is important to acknowledge the challenges and limitations in our study regarding the use of social media data. Firstly, the data collected from social media may not represent a representative sample of the population, as certain groups of people may be underrepresented or excluded.

Moreover, access to data may be limited due to privacy policies and platform restrictions, which can affect the validity and generalizability of the obtained results. Furthermore, quantitative data analysis may provide a partial and superficial understanding of the phenomenon, without thoroughly considering the context and broader meaning of the content posted on social media.

## Data availability statement

The original contributions presented in the study are included in the article/supplementary material, further inquiries can be directed to the corresponding author.

## Author contributions

The author confirms being the sole contributor of this work and has approved it for publication.

## Footnotes

^1^https://www.instagram.com/p/CAH8DMCjJzR/?utm_source=ig_embed&ig_rid=8c94df79-58bb-40b1-ac92-d5cb817809ca
